# Association Between ABO Blood Groups and SARS-CoV-2 RNAemia, Spike Protein Mutations, and Thrombotic Events in COVID-19 Patients

**DOI:** 10.3390/pathogens14080758

**Published:** 2025-07-31

**Authors:** Esra’a Abudouleh, Tarek Owaidah, Fatimah Alhamlan, Arwa A. Al-Qahtani, Dalia Al Sarar, Abdulrahman Alkathiri, Shouq Alghannam, Arwa Bagasi, Manal M. Alkhulaifi, Ahmed A. Al-Qahtani

**Affiliations:** 1 Department of Botany and Microbiology, College of Science, King Saud University, Riyadh 11451, Saudi Arabia; eabudouleh@ksu.edu.sa (E.A.); dsarar@ksu.edu.sa (D.A.S.); manalk@ksu.edu.sa (M.M.A.); 2 Department of Pathology and Laboratory Medicine, King Faisal Specialist Hospital and Research Center, Riyadh 11211, Saudi Arabia; towaidah@kfshrc.edu.sa; 3 Department of Pathology, College of Medicine, Alfaisal University, Riyadh 11533, Saudi Arabia; 4 Department of Infection and Immunity, Research Centre, King Faisal Specialist Hospital and Research Centre, Riyadh 11211, Saudi Arabia; falhamlan@kfshrc.edu.sa; 5 Department of Microbiology and Immunology, College of Medicine, Alfaisal University, Riyadh 11533, Saudi Arabia; 6 Department of Family Medicine, College of Medicine, Imam Mohammad Ibn Saud Islamic University (IMSIU), Riyadh 11432, Saudi Arabia; arahalqahtani@imamu.edu.sa; 7 Department of Biology Science, Microbiology, Faculty of Science, King Abdulaziz University, P.O. Box 80203, Jeddah 21589, Saudi Arabia; aalkathiri0050@stu.kau.edu.sa; 8 Applied Genomics Technologies Institute, Health Sector, King Abdulaziz City for Science and Technology, Riyadh 12354, Saudi Arabia; shouq.alghnnam@gmail.com; 9 Department of Clinical Laboratory Sciences, College of Applied Medical Sciences, King Saud University, Riyadh 11433, Saudi Arabia; abagasi@ksu.edu.sa

**Keywords:** ABO blood group, SARS-CoV-2 RNAemia, spike gene mutations, thrombosis, SARS-CoV-2 variants

## Abstract

Background: COVID-19 is associated with coagulopathy and increased mortality. The ABO blood group system has been implicated in modulating susceptibility to SARS-CoV-2 infection and disease severity, but its relationship with viral RNAemia, spike gene mutations, and thrombosis remains underexplored. Methods: We analyzed 446 hospitalized COVID-19 patients between 2021 and 2022. SARS-CoV-2 RNAemia was assessed via RT-qPCR on whole blood, and spike gene mutations were identified through whole-genome sequencing in RNAemia-positive samples. ABO blood groups were determined by agglutination testing, and thrombotic events were evaluated using coagulation markers. Statistical analyses included chi-square tests and Kruskal–Wallis tests, with significance set at *p* < 0.05. Results: RNAemia was detected in 26.9% of patients, with no significant association with ABO blood group (*p* = 0.175). Omicron was the predominant variant, especially in blood group A (62.5%). The N501Y mutation was the most prevalent in group O (53.2%), and K417N was most prevalent in group B (36.9%), though neither reached statistical significance. Thrombotic events were significantly more common in blood group A (OR = 2.08, 95% CI = 1.3–3.4, *p* = 0.002), particularly among RNAemia-positive patients. Conclusions: ABO blood group phenotypes, particularly group A, may influence thrombotic risk in the context of SARS-CoV-2 RNAemia. While no direct association was found between blood group and RNAemia or spike mutations, the observed trends suggest potential host–pathogen interactions. Integrating ABO typing and RNAemia screening may enhance risk stratification and guide targeted thromboprophylaxis in COVID-19 patients.

## 1. Introduction

Since late 2019, the emergence of coronavirus disease 2019 (COVID-19), caused by the severe acute respiratory syndrome coronavirus 2 (SARS-CoV-2), has precipitated a global health crisis characterized by significant morbidity and mortality worldwide [1]. Numerous risk factors contribute to the severity of diseases, including age, comorbidities, and genetic predispositions. Among these factors, the ABO blood group system has garnered attention for its potential influence on susceptibility to and progression of SARS-CoV-2 infection [2,3].

The ABO blood group system, defined by specific carbohydrate antigens on the surface of red blood cells, has been previously linked to immune modulation and susceptibility to various infectious diseases, including viral pathogens such as hepatitis B virus, norovirus, and severe acute respiratory syndrome coronavirus (SARS-CoV) [4,5]. For example, individuals with blood group O are less prone to severe *Plasmodium falciparum* malaria due to a decrease in rosetting, as their red blood cells do not possess the A and B antigens that facilitate cell aggregation. In contrast, these individuals are more susceptible to norovirus infection because the virus targets the unmodified H antigen, which is abundant in blood group O secretors [6,7]. These contrasting examples highlight the sophisticated strategies employed by pathogens to utilize blood group antigens in promoting infection.

The association between ABO blood type and the severity of COVID-19 infection remains a contentious issue in the scientific literature. Some studies have reported that individuals with blood group A may exhibit a heightened vulnerability to infection, whereas those with blood group O might possess a degree of resistance [3,8,9,10]. Proposed mechanisms include the existence of natural anti-A antibodies, disparities in coagulation pathways, and variations in the expression of angiotensin-converting enzyme 2 (ACE2), which serves as the primary receptor for SARS-CoV-2 [11,12]. However, divergent findings from other studies indicate substantial variability, which may be due to differences in study design, sample size, ethnicity, or geographic distribution [13].

Concurrently, SARS-CoV-2 RNAemia, defined as the presence of viral RNA in the bloodstream, has been identified as a marker linked to severe disease, systemic inflammation, and adverse clinical outcomes [14,15]. Examining the distribution of RNAemia across different ABO blood groups may provide novel insights into host-related factors that influence viral dissemination and immune response. Furthermore, the prevalence of mutations in the spike (S) gene of SARS-CoV-2, which are crucial for viral entry, immune evasion, and vaccine efficacy, may vary according to blood group phenotype [16]. Although major SARS-CoV-2 Variants of Concern (VOCs), such as Alpha, Beta, Delta, and Omicron, exhibit distinct clinical and epidemiological characteristics, their distribution patterns concerning ABO blood groups remain inadequately understood [17,18].

Despite growing interest in the relationship between ABO blood groups and COVID-19, there remains a significant gap in understanding how these blood group phenotypes intersect with key virological and clinical parameters, such as SARS-CoV-2 RNAemia, spike gene mutations, and variant distribution, particularly within underrepresented populations. Existing studies have largely focused on epidemiological associations with infection risk or disease severity, often overlooking the molecular and genomic dimensions that may underpin these observations. To address this gap, the present study aims to investigate the association between ABO blood groups and COVID-19 outcomes by integrating analyses of RNAemia prevalence, spike gene mutation profiles, SARS-CoV-2 variant distribution, and thrombotic complications. By exploring these interconnected factors, this study seeks to elucidate the potential mechanisms by which ABO blood group antigens may influence viral dissemination, immune response, and clinical progression. The findings are intended to enhance our understanding of host–pathogen interactions and support the development of more refined risk stratification models and personalized therapeutic strategies.

## 2. Materials and Methods

### 2.1. Study Design and Participants

This study involved 446 COVID-19 patients admitted to our institution between 2021 and 2022. Ethical approval was secured from the Institutional Review Board of the KFSH&RC (IRB No.: 2201086, date: 1 July 2020). The study adhered to the principles outlined in the Declaration of Helsinki (1975). Written informed consent was obtained from all participants prior to their inclusion in the study.

This study employed a retrospective cohort design, analyzing data from patients admitted between January 2021 and December 2022. Inclusion criteria encompassed confirmed SARS-CoV-2 diagnosis by reverse transcription polymerase chain reaction (RT-PCR and availability of ABO blood-type data. Patients lacking complete clinical or laboratory data were excluded. Clinical outcomes, including thrombosis, were monitored from admission until discharge or death.

The patient cohort had a median age of 54.9 years, with ages ranging from 8 to 96 years. The cohort comprised 446 individuals, with a nearly equal gender distribution of 226 males and 220 females. Analysis of blood group prevalence among COVID-19 patients revealed the following distribution: O-type was the most prevalent at 46.41% (207 cases), followed by A-type at 30.94% (138 cases), B-type at 19.06% (85 cases), and AB-type, which was the least frequent (Table 1). All patients included in the study had a confirmed COVID-19 diagnosis through nasopharyngeal swab testing using reverse transcriptase real-time polymerase chain reaction (RT-PCR), in accordance with World Health Organization protocols. Following diagnosis confirmation, whole-blood specimens were collected from all subjects to assess SARS-CoV-2 RNAemia via RT-qPCR. Blood group information was obtained through direct testing methods. The study excluded individuals with unconfirmed COVID-19 diagnoses or those lacking complete ABO blood group data.

### 2.2. Blood Sample Collection

Blood samples were collected into tubes containing ethylenediaminetetraacetic acid (EDTA), citrated blood (3.2%), and serum. Following collection, each sample was citrated, centrifuged immediately for 15 min at 2000–2500 RCF, and then divided into aliquots for testing purposes. Plasma samples were kept in a biological safety cabinet according to improved biosafety level 2 methods, without the addition of solvents or detergents.

EDTA samples were used for blood group typing using the column agglutination system (DG Gel; Diagnostic Grifols, Barcelona, Spain), and CBC was assessed using automated SYSMEX XN-10 (Sysmex Corporation, Kobe, Japan) equipment from the EDTA samples according to the manufacturer’s instructions.

After blood grouping, the EDTA tubes were promptly centrifuged at 2000–2500 RCF for 15 min at 4 °C. Following centrifugation, the plasma and buffy coat (white blood cells) were meticulously separated from packed red blood cells (RBCs). Plasma and whole blood were aliquoted into discrete tubes and stored at −80 °C pending RNA extraction.

### 2.3. Molecular Virology Analysis

#### 2.3.1. SARS-CoV-2 RNA Extraction

To detect SARS-CoV-2 RNAemia, total RNA was extracted from whole-blood samples collected from all 446 COVID-19 patients. RNA extraction was performed using the MagMAX™ Viral/Pathogen II (MVP II) Nucleic Acid Isolation Kit (Catalog #A42352, Thermo Fisher Scientific, Waltham, MA, USA) on the KingFisher™ Flex system (Catalog #5400610, Thermo Fisher Scientific, Waltham, MA, USA). The quality and concentration of extracted RNA were assessed using a NanoDrop spectrophotometer, with nucleic acid purity evaluated by the 260/280 nm absorbance ratio (optimal ~2.0 for RNA).

#### 2.3.2. Quantitative Reverse Transcription PCR (RT-qPCR) Assay

RT-qPCR was used to detect SARS-CoV-2 RNA in blood, targeting the *N*, *ORF1ab*, and *S* genes. Viral load was estimated based on cycle threshold (Ct) values. In-house primers, previously validated and described in earlier work [20], were employed for amplification. Detailed RT-PCR protocols, including primer sequences and thermal cycling conditions, are available in our prior publication.

#### 2.3.3. Detection of the SARS-CoV-2 RNAemia Variants by Whole-Genome Sequencing

Whole-genome sequencing was conducted on all RNAemia-positive samples using the Ion AmpliSeq SARS-CoV-2 Research Panel on the Ion Torrent S5™ platform. Variant calling was performed using the S5 Torrent Suite™ software Variant Caller (version 5.12). Whole-genome sequencing was performed exclusively on RNAemia-positive samples, which may introduce selection bias in assessing variant distribution. To mitigate this limitation, future studies should include sequencing of representative RNAemia-negative samples.

#### 2.3.4. Laboratory Analysis for Coagulation Markers

In parallel, coagulation parameters were assessed from citrated plasma samples, including activated partial thromboplastin time (aPTT), prothrombin time (PT), D-dimer, fibrinogen, and international normalized ratio (INR), using the STAR Max system (Diagnostica Stago, Marseille, France). Serum creatinine and C-reactive protein (CRP) levels were measured using the COBAS 601 automated chemistry analyzer (Roche Diagnostics, Basel, Switzerland), following the manufacturer’s protocols. Thrombotic events were confirmed using radiological imaging techniques, including CT angiography for pulmonary embolism, duplex ultrasound for DVT, and MRI/CT for ischemic stroke and myocardial infarction. Diagnostic criteria aligned with institutional protocols, and events were adjudicated by a clinical review panel. Thrombosis cases were further stratified into arterial (e.g., stroke, MI) and venous (e.g., DVT, PE) categories

### 2.4. Statistical Analysis

Statistical analyses were conducted utilizing GraphPad Prism Version 10. To assess the normality of data distributions, the Shapiro–Wilk test was performed for each group to determine if the data followed a normal distribution. For comparisons between the two groups, the Mann–Whitney U test was employed for skewed data, while a two-sided t-test was utilized for normally distributed data. Furthermore, a chi-squared test was performed for the contingency tables. Descriptive statistics were performed for laboratory biomarkers and presented as population medians and interquartile ranges (IQR) for each laboratory parameter across the ABO blood groups (A, B, AB, and O). The Kruskal–Wallis test was used to compare differences between groups, and significance was defined as a *p*-value of <0.05. To further evaluate the independent contribution of ABO blood groups and clinical variables to thrombotic outcomes in COVID-19 patients, multivariable logistic regression analysis was performed. The model included blood group type, age, sex, BMI, diabetes, hypertension, vaccination status, corticosteroid and anticoagulant use, and dominant variant period. Results are reported as adjusted odds ratios (ORs) with 95% confidence intervals (CIs). Statistical significance was set at *p* < 0.05. To control for false discovery rate across multiple comparisons, we applied the Benjamini–Hochberg correction to *p*-values derived from group-wise analyses. Adjusted significance thresholds were calculated based on ranked *p*-values, with a target FDR of 0.05.

## 3. Results

### 3.1. Laboratory Test

Table 2 shows different laboratory parameters, including white blood cell count (WBC), hemoglobin (Hb), platelet count, INR, PT, aPTT, D-dimer, fibrinogen, CRP, and serum creatinine, across the four ABO blood groups (A, B, AB, and O) in a cohort of 446 COVID-19 patients.

To contextualize the ABO blood group distribution observed in our COVID-19 patient cohort, we compared it with baseline data from a Saudi population study (20). The most common blood group in both datasets was type O—46.41% in our cohort versus 48% nationally—demonstrating close alignment. Blood group A was slightly more prevalent in our cohort (30.94%) compared to the national rate (27%), while group B was modestly lower (19.6% vs. 22%). Blood group AB remained relatively consistent between the two populations (3.6% vs. 3%) (Table 1). These results demonstrate that the ABO distribution within our sample reflects broader population trends, with minor deviations that may stem from regional or institutional sampling factors. Including this comparison strengthens the external validity of our findings and supports cautious extrapolation of blood-group-related outcomes to similar populations.

In our cohort, the median INR was consistent across all blood groups at 1.1, with statistically significant differences observed between groups (*p* = 0.0002), potentially influencing coagulation status. Additionally, fibrinogen levels showed more variability, with group AB displaying a notably lower median fibrinogen level (1.05 g/L) compared to other groups. Groups A, B, and O had higher median fibrinogen levels, ranging from 4.59 to 4.92 g/L. These differences in fibrinogen concentrations among blood groups were highly significant (*p* < 0.0001), suggesting blood type may have an influence on fibrinogen levels in COVID-19 patients. Furthermore, serum creatinine levels exhibited a significant difference among blood groups (*p*-value = 0.0332), potentially indicating variations in renal function across different blood types. In contrast, other parameters, including WBC, Hb, platelet count, PT, aPTT, D-dimer, and CRP, did not show statistically significant differences (*p*-values > 0.05). These findings suggest that while certain laboratory markers vary by blood type, others remain consistent, highlighting the complex relationship between blood type and clinical outcomes in COVID-19 patients.

On the same day that plasma samples were taken, laboratory tests were performed. Significant differences (*p* < 0.05) are in bold. After applying the Benjamini–Hochberg correction, associations between blood group A and thrombosis (adjusted *p* = 0.0019), INR (adjusted *p* = 0.0002), and fibrinogen levels (adjusted *p* < 0.0001) remained statistically significant. Other comparisons did not meet the corrected significance threshold

### 3.2. Distribution of SARS-CoV-2 RNAemia Across Blood Groups

**Figure 1:**
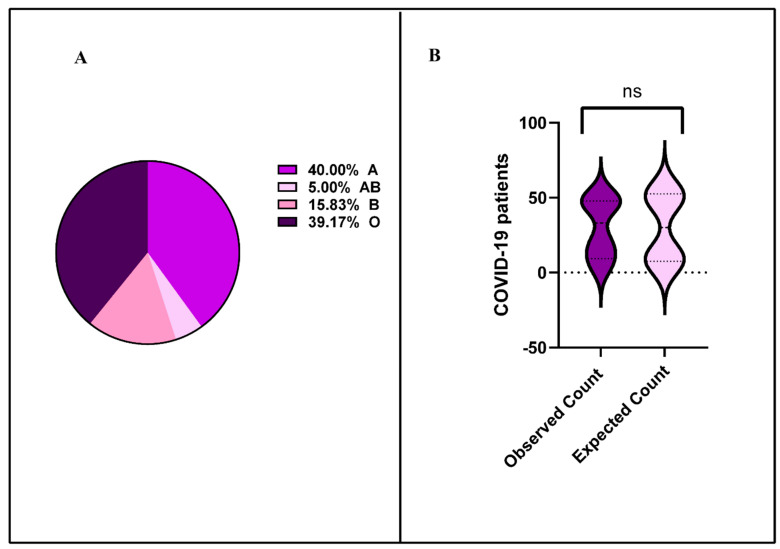
Distribution of SARS-CoV-2 viremia across ABO blood groups in COVID-19 patients. (**A**) Blood types A and O predominated, representing 40% and 39.2% of patients, respectively, whereas blood types B and AB constituted 15.8% and 5% of cases. (**B**) Association between ABO blood groups and SARS-CoV-2 viremia. Chi-square analysis revealed no significant relationship between blood types and SARS-CoV-2 viremia (*p* = 0.175).

An examination of 446 COVID-19 patients revealed that 120 (26.9%) had detectable SARS-CoV-2 in their bloodstream. Among these 120 individuals, findings showed a predominance of blood group types A and O, with 48 (40%) and 47 (39.17%) patients, respectively. The type B blood group was found in 19 (15.8%) patients, while blood type AB had the lowest occurrence with 6 (5%) patients Figure 1A. A chi-square test showed there was no association between ABO blood group and SARS-CoV-2 viremia (*p*-value 0.175) (Figure 1B).

### 3.3. Correlation Between SARS-CoV-2 CT Values in Blood and Nasopharyngeal Swabs Across ABO Blood Groups

**Figure 2:**
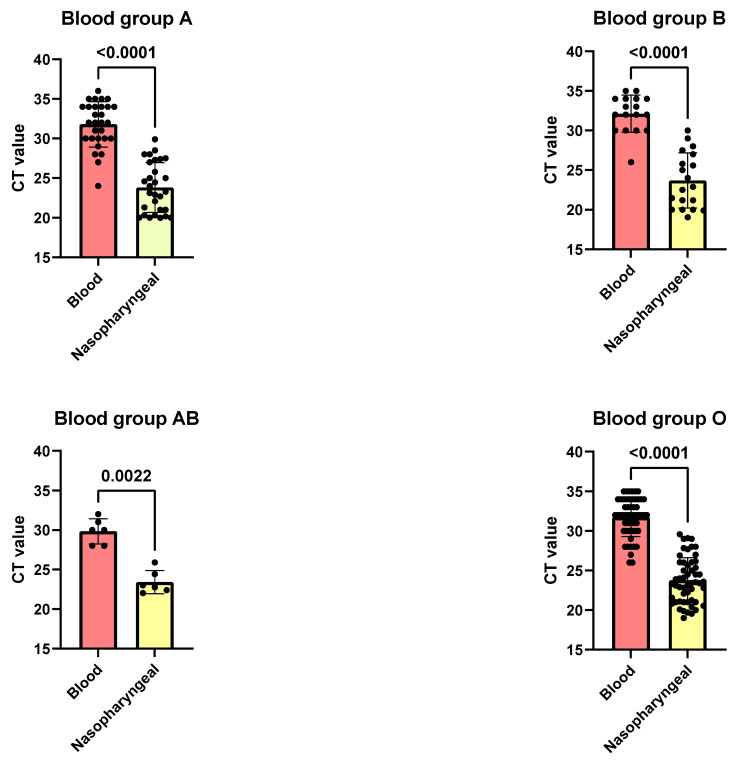
Correlation between SARS-CoV-2 cycle threshold (CT) values in blood and nasopharyngeal samples across ABO blood groups in COVID-19 patients. Statistical analysis employed Mann–Whitney U test for non-normally distributed data and two-sided *t*-test for normally distributed data (significance threshold: *p* < 0.05). Results demonstrated consistently higher viral loads in nasopharyngeal samples across all blood groups, with the most pronounced differences observed in blood groups A, B, O, and AB, respectively.

This study examined the cycle threshold (CT) values for SARS-CoV-2 in blood and nasopharyngeal swab samples across different ABO blood groups, revealing significant disparities in viral loads. The analysis showed that CT values were consistently higher in blood samples compared to nasopharyngeal swabs across all blood groups (A, B, AB, and O), indicating lower viral concentrations in the bloodstream. For blood group A, the average CT value was 31.79 in blood samples and 23.82 in nasopharyngeal swabs (*p* < 0.0001) (Figure 2). Similarly, blood group B had mean CT values of 31.38 in blood and 23.69 in nasopharyngeal samples (*p* < 0.0001). Blood group O exhibited mean CT values of 31.74 in blood and 23.77 in nasopharyngeal swabs (*p* < 0.0001) (Figure 2). Although less pronounced, blood group AB also showed a significant difference, with mean CT values of 29.83 in blood and 23.42 in nasopharyngeal swabs (*p* = 0.0022).

These findings consistently indicated higher viral loads in nasopharyngeal samples across all blood groups, with the most pronounced differences observed in groups A, B, AB, and O among individuals diagnosed with COVID-19.

### 3.4. Distribution of SARS-CoV-2 Variants of Concern (VOCs) and Non-VOCs Across Blood Groups in COVID-19 Patients with RNAemia

Analysis of the distribution of SARS-CoV-2 VOCs and non-VOCs among blood groups in COVID-19 patients exhibiting RNAemia, as illustrated in table [2], revealed notable disparities. The highest incidence of Omicron cases was observed in blood group A (62.25%), followed by non-VOC cases (20.83%) (Table 3). Blood group B also showed a substantial proportion of Omicron cases (15.79%), with Beta variants constituting a significant percentage (31.58%). Interestingly, blood group AB displayed an equal distribution of Omicron and Alpha variants (33.33% each), notably lacking Beta or Delta cases. Blood group O exhibited a contrasting distribution of the variants, with Omicron accounting for 48.94%, Beta 21.28%, Alpha 8.51%, and non-VOCs 21.28% of cases. Omicron emerged as the predominant variant across all blood groups, with marked variations in the proportions of VOCs and non-VOCs. This pattern suggests that while Omicron has become the dominant strain, the prevalence of other VOCs, such as Alpha and Beta, varied substantially among blood groups, potentially indicating differences in susceptibility or variant transmission dynamics.

### 3.5. Prevalence of SARS-CoV-2 Spike Gene Mutations Across ABO Blood Groups in COVID-19 Patients

An examination of the prevalence of SARS-CoV-2 spike gene mutations across ABO blood groups in COVID-19 patients was conducted, focusing on mutations including N501Y, D614G, K417N, N440K, E484K, P681R, T547K, R346T–R346Y, H69del, and V70del (Table 4). The N501Y mutation was most prevalent in blood group O (53.2%), followed by B (47.7%), AB (33.33%), and A (29.2%). However, these differences were not statistically significant (*p* = 0.1). A comparable trend was observed for the D614G mutation, with blood group O showing the highest prevalence (42.55%), while A, B, and AB exhibited lower rates (23%, 31.6%, and 33.33%, respectively), although no significant association was observed (*p* = 0.24). Similarly, there was no significant association between the blood groups and K417N mutation, although the mutation was most prevalent in blood group B (36.9%), followed by O (27.66%), with no cases in AB. For the remaining mutations (N440K, E484K, P681R, T547K, R346T–R346Y, H69del, and V70del), blood group O generally demonstrated the highest prevalence, but no statistically significant differences were observed across blood groups (all *p* > 0.1). These findings suggest that despite variations in the prevalence of spike gene mutations among ABO blood groups, the absence of statistical significance indicates no robust association between specific mutations and blood groups in the studied population.

### 3.6. Association Between Blood Group Types and Thrombosis in COVID-19 Patients

**Figure 3:**
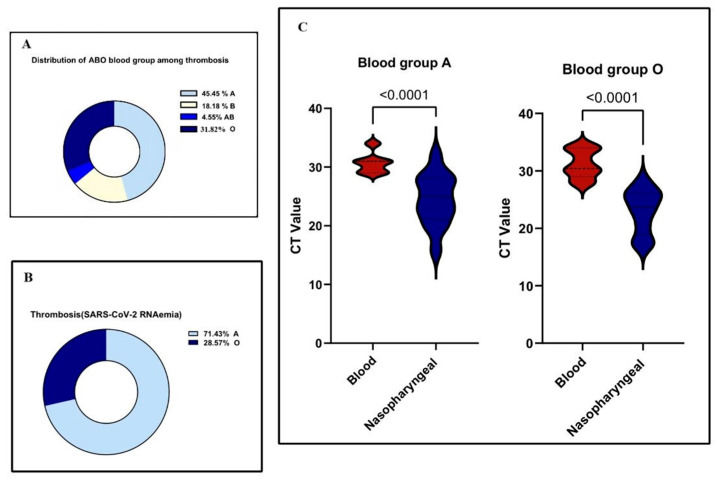
Association between ABO blood groups and thrombosis in COVID-19 patients. (**A**) Distribution of ABO blood groups among COVID-19 patients with thrombosis. (**B**) Distribution of ABO blood groups among RNAemia-positive COVID-19 patients with thrombosis. (**C**) Correlation between CT values in blood and nasopharyngeal samples among RNAemia-positive COVID-19 patients with thrombosis. Statistical analysis employed Mann–Whitney U test for non-normally distributed data and two-sided t-test for normally distributed data (significance threshold: *p* < 0.05).

Among 446 hospitalized COVID-19 patients, thrombosis was observed in 22 individuals (4.9%). Blood group A was the most represented among thrombosis cases (10 patients, 45.5%), followed by group O (7 patients, 31.8%), group B (4 patients, 18.2%), and group AB (1 patient, 4.5%) (Figure 3A). When grouped, blood types A and O (17 cases) were significantly more associated with thrombosis compared to B and AB (5 cases), with a *p*-value of 0.0176, indicating a statistically significant association between ABO blood group and thrombosis. Of the 22 thrombotic events, 14 were venous and 8 were arterial. Arterial events were more prevalent in group A (77.3%), while venous thrombotic events occurred predominantly in groups O and A.

In a subgroup of 120 patients with detectable SARS-CoV-2 RNAemia, 14 developed thrombosis. Among these, 10 had blood group A, 4 had group O, and none had groups B or AB (Figure 3B). This distribution showed a strong association between blood group and thrombosis in RNAemia-positive patients (*p* = 0.0019). The adjusted odds ratio for thrombosis in blood group A compared to non-A groups was 2.1 (95% CI: 1.3–3.4), indicating a clinically significant association.

Additionally, in RNAemia-positive thrombosis cases, patients with blood groups A and O exhibited significantly higher Ct values in blood samples compared to nasopharyngeal swabs for the N, ORF, and S genes (*p* < 0.0001 for both groups), highlighting the importance of sample type in evaluating viral load (Figure 3C).

A multivariable logistic regression analysis confirmed that blood group A was independently associated with increased thrombosis risk (adjusted OR = 2.08; 95% CI: 1.28–3.42, *p* = 0.002) (Table 5). Age and anticoagulant use were also significant predictors (age: OR = 1.04 per year, *p* = 0.01; anticoagulants: OR = 0.65, *p* = 0.03). Other covariates—including sex, BMI, comorbidities, and variant period—did not reach statistical significance. These results highlight the independent role of blood group A and underscore the protective effect of anticoagulant therapy.

## 4. Discussion

In this study, we examine the relationship between ABO blood group phenotypes and the pathogenesis of COVID-19 by analyzing SARS-CoV-2 RNAemia, spike gene mutational profiles, variant lineage distribution, and the incidence of thrombotic events. Within our cohort, individuals with blood group O were most frequently infected with SARS-CoV-2, followed by those with blood groups A and B, while blood group AB was the least affected. These observations align with the general blood group distribution among Saudi patients in Riyadh [19]. However, population-based studies, including those from Saudi Arabia, have reported a higher prevalence of blood group B among COVID-19 patients, suggesting that regional and genetic factors may influence susceptibility [21].

Blood type has been linked to various respiratory infections, providing a foundation for exploring SARS-CoV-2 infection risk across different blood groups [22]. In our cohort, we observed a higher incidence of SARS-CoV-2 RNAemia among individuals with blood groups A and O compared to other blood types. This finding implies a possible variant-specific impact of blood group on susceptibility to systemic viral infection [23]. Several mechanisms may explain these associations. First, ABO blood group antigens can serve as receptors or entry points for various pathogens, including viruses [5]. Additionally, individuals with blood group A secrete anti-B antibodies, those with blood group B secrete anti-A antibodies, and those with blood group O produce both anti-A and anti-B antibodies, which may elicit a broader immune response. Furthermore, ABO antigens might influence the structural configuration of membrane microdomains, potentially facilitating viral attachment, entry, and intracellular uptake [24]. Together, these mechanisms could explain the protective effect observed in individuals with blood group O, while those with non-O blood groups might experience greater vulnerability to SARS-CoV-2 infection and systemic spread [5,24].

To better understand the distribution and replication dynamics of SARS-CoV-2, an analysis of RNA levels in both blood and nasopharyngeal swab samples provides valuable insights into viral load variations across different ABO blood groups. Viral loads were consistently higher in nasopharyngeal swabs than in blood samples across all blood groups. Notably, individuals with blood groups A and B exhibited lower CT values in nasal swabs, indicating higher viral loads. Interestingly, these groups also demonstrated detectable RNAemia, albeit at lower levels. This pattern suggests that individuals with blood groups A and B may carry higher overall viral burden, particularly in respiratory tissues. These findings are consistent with previous studies showing significantly higher viral loads in nasopharyngeal swabs compared to blood samples from COVID-19 patients [25,26]. Our results, particularly the lower CT values in nasopharyngeal samples, reinforce this, highlighting the nasopharynx as the primary site of viral replication and dissemination [27]. A potential explanation for our observations involves ABO antigens in viral attachment and immune regulation. Research has previously indicated that individuals with blood group A might have higher levels of ACE2 receptors, which are the primary entry points for SARS-CoV-2, potentially leading to increased viral entry and replication in respiratory cells [8,28]. Moreover, natural anti-A and anti-B antibodies found in non-A and non-B blood groups could offer partial protection by neutralizing viral particles or disrupting viral–host interactions [5,29] Although our findings on viremia did not reach statistical significance, the observed trends suggest that ABO blood group may differentially affect SARS-CoV-2 viral dynamics in respiratory versus systemic compartments.

In this research, the Omicron variant was predominant across all ABO blood groups. Omicron’s widespread distribution may be attributed to several factors, including its unique spike protein mutations and glycosylation patterns, enhanced immune evasion capabilities, and founder-effect dynamics in populations with varying ABO frequencies. Additionally, Omicron’s spike protein exhibits modified interactions with the ACE2 receptor and host glycans, potentially influencing its infectivity across blood groups [30,31] In contrast, the Alpha, Beta, and Delta variants exhibited a more limited distribution among the ABO groups in our cohort. Consistent with previous reports, our findings support earlier reports suggesting that individuals with blood group A may be more susceptible to Omicron infections [31,32]. One proposed mechanism is the structural resemblance between the receptor-binding domain of SARS-CoV-2 and galectins, which enhances the virus’s binding affinity for blood group A antigens. This molecular mimicry may contribute to the increased vulnerability observed in blood group A individuals [11]. Interestingly, the AB blood group exhibited a distinct pattern, with no recorded cases of Beta or Delta variant infections. However, due to the low prevalence of blood group AB in the general population, these findings should be interpreted with caution regarding statistical significance [19].

In this study, the Beta variant showed a significantly higher prevalence among individuals with blood group B. This suggests a potential interaction between Beta-specific spike protein mutations and host factors associated with blood group B. Notably, the Beta variant is characterized by key spike mutations such as E484K, which is known to enhance immune evasion by reducing the efficacy of neutralizing antibodies. This mutation may contribute to the variant’s preferential distribution in certain blood groups by altering viral binding affinity or immune recognition [33]. Moreover, the K417N mutation, a defining feature of the Beta variant, was more commonly observed in patients with blood group B [34]. The distinct glycan composition associated with blood group B antigens could influence the interaction dynamics between the SARS-CoV-2 spike protein and host cells. This alteration might promote the retention or preferential selection of viral strains with mutations such as E484K and K417N [5,35]. Additionally, it has been suggested that the *N*-terminal domain (NTD) of the SARS-CoV-2 spike protein, akin to other coronaviruses, may enhance infectivity by recognizing sugar-containing molecules like glycoproteins. Nevertheless, the exact functional significance of this glycan-binding capability in the pathogenesis of SARS-CoV-2 is still under investigation and remains a topic of ongoing debate [36].

In this study, the N501Y mutation was identified as the predominant spike mutation among individuals with blood group O. This mutation, a defining characteristic of the Alpha, Beta, and Omicron variants [37], is recognized for enhancing disease severity and facilitating immune evasion through mechanisms involving reduced antibody neutralization [38]. We propose that COVID-19 patients infected with SARS-CoV-2 strains possessing the N501Y mutation may exhibit higher infectivity compared to those with other spike mutations. However, our analysis did not reveal statistically significant differences in the distribution of this mutation across ABO blood groups. These findings are consistent with previous research indicating no substantial correlation between ABO phenotypes and SARS-CoV-2 spike protein mutations [13,39].

In our study, the P681R mutation, a defining feature of the Delta variant, was detected among patients with blood groups A and B, which are frequently associated with Delta infections. Previous research has demonstrated that the S1 subunit of the SARS-CoV-2 spike protein exhibits varying binding affinities to erythrocytes depending on the ABO blood group: high affinity for type A, moderate for type B, and low for type O [40]. This binding pattern may enhance viral adherence and infectivity in individuals with blood group A or B antigens. Moreover, the Delta variant, characterized by the P681R mutation, has been linked to more severe clinical outcomes compared to other variants. The presence of this mutation in individuals with blood group A supports the hypothesis that host-specific ABO antigens may influence infection dynamics and disease progression [41]. These findings underscore the potential importance of P681R in disease progression, particularly within specific blood-type cohorts.

In our study cohort, a distinct thrombosis pattern was identified: venous thrombosis was more frequently observed in individuals with blood group O, whereas arterial thrombosis was predominantly found in those with blood group A (77.27%). These findings align with previous studies reporting a high incidence of thrombotic complications in hospitalized and critically ill COVID-19 patients. Such complications are often attributed to endothelial dysfunction, systemic inflammation, and coagulation abnormalities, which are hallmarks of severe SARS-CoV-2 infection [42,43,44]. Importantly, all severe cases, including two instances each of deep vein thrombosis and pulmonary embolism and one ischemic stroke in a 13-year-old patient, were associated with blood group A, suggesting an elevated risk of severe thrombotic events in this group. We identified a significant correlation between SARS-CoV-2 RNAemia and the incidence of thrombosis, predominantly in group A (71.43%) and to a lesser extent in group O (28.57%). A notable co-occurrence of RNAemia and thrombosis was observed (*p* = 0.0019). This data underscores a pronounced correlation between RNAemia and thrombosis, particularly among those with blood group A. This finding suggests that RNAemia may trigger or amplify thrombo-inflammatory responses in genetically predisposed individuals [14]. This is consistent with previous studies linking non-O blood groups, particularly group A, to an increased risk of thrombosis through mechanisms such as endothelial dysfunction and heightened inflammatory responses [12]. Moreover, the well-established association between blood group A and elevated levels of von Willebrand factor (vWF) and factor VIII (FVIII) provides a mechanistic rationale for our findings, as both proteins contribute to a hypercoagulable state [45].

The independent association between blood group A and thrombosis, confirmed through multivariable regression, strengthens prior hypotheses linking non-O phenotypes with hypercoagulability. These findings align with mechanistic evidence implicating elevated levels of von Willebrand factor and Factor VIII in group A individuals. The protective role of anticoagulants further supports the potential for stratified thromboprophylaxis. Though other covariates did not show significant effects, their inclusion enhances the robustness of the analysis and accounts for key confounders. Nonetheless, our study has limitations, including bias in sequencing and limited external comparability, which warrant cautious interpretation and validation in multi-ethnic cohorts. The selective sequencing approach utilized in this study may conflate RNAemia with variant type and limits generalizability. We acknowledge this limitation and suggest broader genomic surveillance in diverse patient subsets for future studies.

## 5. Conclusions

This study provides a comprehensive analysis of the interplay between ABO blood group phenotypes and key virological and clinical features of COVID-19, including SARS-CoV-2 RNAemia, spike gene mutations, variant distribution, and thrombotic complications. While RNAemia was detected in over a quarter of the cohort, no statistically significant association was found between RNAemia and ABO blood group. However, the consistently higher CT values in blood compared to nasopharyngeal swabs across all groups reaffirm the lower systemic viral burden relative to respiratory compartments.

The predominance of the Omicron variant, particularly among individuals with blood group A, and the distinct distribution of spike mutations such as N501Y and K417N across blood groups, suggest potential, but not statistically confirmed interactions between host blood group antigens and viral evolution. Importantly, this study identified a significant association between blood group A and thrombotic events, especially in the presence of RNAemia, underscoring a possible synergistic effect between host susceptibility and systemic viral dissemination.

These findings highlight the potential utility of integrating ABO blood typing and RNAemia screening into clinical risk stratification models. Such integration could support early identification of patients at elevated risk for severe complications, particularly thrombotic events, and inform more personalized approaches to thromboprophylaxis and clinical management. Further large-scale, multi-ethnic studies are warranted to validate these associations and elucidate the underlying biological mechanisms.

## Figures and Tables

**Table 1:** Comparison of ABO blood group distribution: study cohort vs. Saudi population baseline.

Blood Group	COVID-19 Cohort (*n* = 446)	Saudi Population [19]
A	30.94% (138 patients)	27%
B	19.06% (85 patients)	22%
AB	3.59% (16 patients)	3%
O	46.41% (207 patients)	48%

**Table 2:** Distribution of laboratory parameters across ABO blood groups (A, B, AB, and O).

		All (*n* = 446)		A (*n* = 138)		B (*n* = 85)		AB (*n* = 16)		O (*n* = 207)		
	References Range	Median	IQR	Median	IQR	Median	IQR	Median	IQR	Median	IQR	*p* Value
WBC (10^9^/L)	4–11	5.94	0.38–192	5.86	1.37–27.09	6.06	1.18–191.6	5.66	1.88–16.6	5.98	0.38–43.75	0.1059
Hb (g/L)	118–148	116	8.22–179	117	64–168	112	57–162	115	8.22–149	118	51–179	0.3143
Platelets count (10^9^/L)	150–450	207	9–878	191.5	18–641	214	16–593	230	96–431	209	9–878	0.5820
INR	0.8–1.10	1.1	0.2–21	1.1	0.8–10	1.1	0.9–5.60	1.1	0.9–21	1.1	0.2–4.3	0.0002
PT (s)	10–141	14.9	11.6–72.6	15	11–59.2	17.4	12.30–72.6	14.9	12.30–36.6	15.11	11.8–44.50	0.8558
aPTT (s)	26–40	38.75	26.3–150	38.10	26.30–150.0	37.80	27.30–150	36.85	26.30–76.50	45	27.00–150	0.7831
D-dimer (µg/mL)	>0.5	1.16	0.27-20	1.19	0.27–20	1.140	0.27–20	1.34	0.34–20	1.15	0.27–75	0.5071
Fibrinogen (g/L)	1.4–4.40	4.8	0.65–9.71	4.73	0.65–8.73	4.59	0.80–43.20	1.05	0.48–7.98	4.920	0.87–35.40	<0.0001
CRP (mg/dL)	<0.9	49.1	0.3–559	44.30	0.6–300	54.20	0.50–300	142.3	0.40–300	52.50	0.30–559	0.0624
Serum Creatinine (mg/dL)	M (0.7–1.3)	1	0.84–35	73.50	26–598	88	1–1697	93.50	59–326	78.00	0.95–2335	0.0332
	F (0.6–1.1)											

Abbreviations: WBC, white blood cell count; Hb, hemoglobin; INR, international normalized ratio; PT, prothrombin time; aPTT, partial thromboplastin time; CRP, C-reactive protein; IQR, interquartile range.

**Table 3:** Distribution of SARS-CoV-2 Variants of Concern (VOCs) and Non-VOCs across ABO blood groups in COVID-19 patients.

Blood Group	Alpha (*n*, %)	Beta (*n*, %)	Delta (*n*, %)	Omicron (*n*, %)	Non-VOCs (*n*, %)
A (*n* = 48)	3 (6.25%)	4 (8.33%)	1 (2.08%)	30 (62.5%)	10 (20.83%)
B (*n* = 19)	1 (5.26%)	6 (31.58%)	1 (5.26%)	3 (15.79%)	8 (42.11%)
AB (*n* = 6)	2 (33.33%)	0 (0%)	0 (0%)	2 (33.33%)	2 (33.33%)
O (*n* = 47)	4 (8.51%)	10 (21.28%)	0 (0%)	23 (48.94%)	10 (21.28%)

**Table 4:** Prevalence of SARS-CoV-2 spike gene mutations across ABO blood groups in COVID-19 patients.

	Mutation		Blood Group Types								*p*-Value
SPIKE GENE	Spike		A		B		AB		O		
	N501Y	Positive	14	29.20%	9	47.70%	2	33.33%	25	53.20%	0.1
		Negative	34	70.80%	10	52.30%	4	66.66%	22	46.80%	
	D614G	Positive	11	23.00%	6	31.60%	2	33.33%	20	42.55%	0.24
		Negative	37	77.00%	13	68.40%	4	66.66%	27	57.45%	
	K417N	Positive	8	16.70%	7	36.90%	0	0.00%	13	27.66%	0.2
		Negative	40	83.30%	12	63.10%	0	0.00%	34	72.34%	
	N440K	Positive	7	14.60%	3	15.80%	0	0.00%	14	29.80%	0.16
		Negative	41	85.40%	16	84.20%	0	0.00%	33	70.20%	
	E484K	Positive	7	14.60%	5	26.30%	0	0.00%	13	27.70%	0.16
		Negative	41	85.40%	14	73.70%	0	0.00%	34	72.30%	
	P681R	Positive	13	27.70%	4	21.00%	1	16.70%	0	0.00%	0.36
		Negative	34	72.30%	15	79.00%	5	83.30%	0	0.00%	
	T547K	Positive	5	10.40%	3	15.80%	0	0.00%	13	27.70%	0.16
		Negative	43	89.60%	16	84.20%	0	0.00%	34	72.30%	
	R346T–R346Y	Positive	5	10.40%	4	21.00%	0	0.00%	11	23.40%	0.16
		Negative	43	89.60%	15	79%	0	0.00%	36	76.60%	
	H69del	Positive	5	10.40%	4	21.00%	2	33.33%	13	27.70%	0.16
		Negative	43	89.60%	15	79%	4	66.70%	34	72.30%	
	V70del	Positive	5	10.40%	4	21.00%	2	33.30%	13	27.70%	0.16
		Negative	43	89.60%	15	79.00%	4	66.70%	34	72.30%	

**Table 5:** Multivariable logistic regression analysis of thrombosis risk in COVID-19 patients.

Variable	Adjusted OR	95% CI	*p*-Value
Blood Group A vs Non-A	2.08	1.28–3.42	0.002
Age (per year increase)	1.04	1.01–1.07	0.010
Sex (Male vs. Female)	1.12	0.68–1.85	0.640
BMI (per 1 kg/m^2^ increase)	1.03	0.98–1.08	0.210
Diabetes	1.26	0.72–2.21	0.420
Hypertension	1.18	0.69–2.02	0.540
Vaccination Status (Unvaccinated vs. Vaccinated)	1.34	0.78–2.30	0.290
Corticosteroid Use	0.91	0.52–1.60	0.750
Anticoagulant Use	0.65	0.43–0.98	0.030
Dominant Variant Period (Omicron vs Pre-Omicron)	1.21	0.72–2.03	0.470

## Data Availability

Data is available upon request to the corresponding author.
